# Development of a Radiomic-Based Model Predicting Lymph Node Involvement in Prostate Cancer Patients

**DOI:** 10.3390/cancers13225672

**Published:** 2021-11-12

**Authors:** Vincent Bourbonne, Vincent Jaouen, Truong An Nguyen, Valentin Tissot, Laurent Doucet, Mathieu Hatt, Dimitris Visvikis, Olivier Pradier, Antoine Valéri, Georges Fournier, Ulrike Schick

**Affiliations:** 1 Radiation Oncology Department, University Hospital, 29200 Brest, France; olivier.pradier@chu-brest.fr (O.P.); ulrike.schick@chu-brest.fr (U.S.); 2 LaTIM, UMR 1101, INSERM, University Brest, 29200 Brest, France; vincent.jaouen@imt-atlantique.fr (V.J.); truong-an.nguyen@chu-brest.fr (T.A.N.); hatt@univ-brest.fr (M.H.); visvikis@univ-brest.fr (D.V.); antoine.valeri@univ-brest.fr (A.V.); georges.fournier@chu-brest.fr (G.F.); 3 IMT Atlantique, 29200 Brest, France; 4 Urology Department, University Hospital, 29200 Brest, France; 5 Radiology Department, University Hospital, 29200 Brest, France; valentin.tissot@chu-brest.fr; 6 Pathology Department, University Hospital, 29200 Brest, France; laurent.doucet@chu-brest.fr

**Keywords:** lymph node involvement, prostate cancer, prediction, radiomics, MRI

## Abstract

**Simple Summary:**

In patients with prostate cancer, lymph node involvement is a risk factor of relapse. Current guidelines recommend extended lymph node dissection to better stage the disease. However, such a surgical procedure is associated with a higher morbidity than limited lymph node dissection. A better selection of patients is thus essential. Radiomics features are quantitative features automatically extracted from medical imaging. Combining clinical and radiomics features, a machine learning-based model seemed to provide added predictive performance compared to state of the art models regarding the risk prediction of lymph-node involvement in prostate cancer patients.

**Abstract:**

Significant advances in lymph node involvement (LNI) risk modeling in prostate cancer (PCa) have been achieved with the addition of visual interpretation of magnetic resonance imaging (MRI) data, but it is likely that quantitative analysis could further improve prediction models. In this study, we aimed to develop and internally validate a novel LNI risk prediction model based on radiomic features extracted from preoperative multimodal MRI. All patients who underwent a preoperative MRI and radical prostatectomy with extensive lymph node dissection were retrospectively included in a single institution. Patients were randomly divided into the training (60%) and testing (40%) sets. Radiomic features were extracted from the index tumor volumes, delineated on the apparent diffusion coefficient corrected map and the T2 sequences. A ComBat harmonization method was applied to account for inter-site heterogeneity. A prediction model was trained using a neural network approach (Multilayer Perceptron Network, SPSS v24.0©) combining clinical, radiomic and all features. It was then evaluated on the testing set and compared to the current available models using the Receiver Operative Characteristics and the C-Index. Two hundred and eighty patients were included, with a median age of 65.2 y (45.3–79.6), a mean PSA level of 9.5 ng/mL (1.04–63.0) and 79.6% of ISUP ≥ 2 tumors. LNI occurred in 51 patients (18.2%), with a median number of extracted nodes of 15 (10–19). In the testing set, with their respective cutoffs applied, the Partin, Roach, Yale, MSKCC, Briganti 2012 and 2017 models resulted in a C-Index of 0.71, 0.66, 0.55, 0.67, 0.65 and 0.73, respectively, while our proposed combined model resulted in a C-Index of 0.89 in the testing set. Radiomic features extracted from the preoperative MRI scans and combined with clinical features through a neural network seem to provide added predictive performance compared to state of the art models regarding LNI risk prediction in PCa.

## 1. Introduction

With 335,230 new estimated cases/year and 69,945 estimated deaths in 2020 in Europe alone [1], prostate cancer (PCa) is the most common cancer among men in western countries. Lymph Node Involvement (LNI) is one of the main prognostic factors and extensive lymph node dissection (eLND) is now the gold standard in patients with high-risk disease operated on for patients as they are at high risk of LNI [2]. However, eLND is associated with substantial morbidity [3,4,5,6,7]. The decision to perform LND in clinically LNI-free patients (cN0) is guided by the probability of nodal metastases, with several models developed for the prediction of LNI risk: the National Comprehensive Cancer Network (NCCN) chooses 2% as a cut-off according to a nomogram developed by the Memorial Sloan Kettering Cancer Centre, whereas the European Association of Urology (EAU) guidelines [8] recommends it for patients with a probability of LNI over 5% based on 2012-Briganti nomogram. Several clinical models were presented for LNI risk prediction [9,10,11,12,13,14]. Magnetic Resonance Imaging (MRI) and its implementation in the daily routine considerably changed both staging and diagnostic management [15]. Lately, Gandaglia et al. developed a new promising algorithm using qualitative data from MRIs in patients with targeted biopsies [16]. Even if targeted combined with systematic biopsies provide a better assessment of the Gleason score [17], software-based fusion targeted biopsies require special logistics and a certain cost [18], which explains its under-utilization compared to cognitive fusion, leaving place for better LNI risk assessment tools.

Such a better patients’ selection could benefit the patients undergoing surgery with a reduction in unnecessary eLND for selected patients.

Quantitative analysis of medical images such as MRIs has shown to reflect macroscopic tumoral heterogeneity and has been implemented in several prediction and prognostic models, especially in PCa [19,20]. However, the added value of radiomic features on LNI prediction has never been studied.

Machine learning methods, especially artificial neural networks (NN) [21], have the potential to efficiently model the synergistic interaction between variables using a flexible nonlinear relationship.

In this report, we aimed to develop and internally validate a NN radiomic-based LNI prediction model.

## 2. Materials and Methods

### 2.1. Population

Patients who underwent a radical prostatectomy and eLND for a PCa in a single institution (University Hospital of Brest) between 2010 and 2018 were retrospectively considered using the pathology database.

Radical prostatectomy and eLND were performed following international guidelines^8^ by a single trained surgeon (GF) [6,22], with ~35 years of experience. Patients with a delay between the preoperative MRI and the surgery superior to 6 months or an MRI unfit for analysis (i.e., missing sequence or slice, hip replacement) were excluded. Patients with no index lesion according to PIRADS V2.1 [23] score after central review by a single specialized radiologist (V.T) with ~15 years of experience.

### 2.2. Prostate MRI

Two different MRI scanners were used: a 3T Phillips (Philips Healthcare, Eindhoven, The Netherlands) and a 1.5T Siemens (Siemens Healthcare, Malvern, PA, USA). Acquisition was performed in accordance with the European Society of Urogenital Radiology (ESUR) guidelines [23]. Full protocol was previously published and summarized in Supplementary Table S1 [19]. 

### 2.3. Clinical and Radiomics Features

Regarding clinical features, we retrospectively collected the preoperative PSA level, the age at the time of surgery, the overall Gleason score along with the primary and secondary biopsy Gleason grades, the number of performed and positive biopsy cores (and rate of positive biopsy cores), the percentage of positive biopsy cores with highest-grade PCa, the percentage of positive biopsy cores with lower grade PCa, the clinical and MRI tumor stages and the NCCN risk classification. When targeted biopsies were performed, we merged the two biopsy techniques, the maximum number of positive cores being set to two by sextant. The PIRADS V2.1 [23] and MRI tumor stage were also collected.

The index lesion was semi-automatically delineated by a single expert (V.B.) using the Fast GrowCut tool imbedded in 3D Slicer©. Full detail regarding the extraction of radiomic features is detailed in Supplementary Protocol 1. As a result, 8651 features (11 clinical and 120 × 4 (fixed bin value) × 9 (original + 8 wavelet filters) × 2 (MRI sequences) = 8640 radiomic features) were available for each patient.

### 2.4. Model Building

The model was built on the training set alone consisting of approximately 60% of the overall cohort. It was then applied on the rest of the patients, defining the testing set (40%). All preselected clinical and radiomic features were then considered for the New-Combined model building, via a Neural Network approach. It was further compared to available nomograms (Partin [9], Yale [11], Roach [10], Briganti 2012 [12], Briganti 2017 [13] and MSKCC [14]). Evaluation of the trained model was carried out using the Area Under the Curve (AUC) along with the C-Index, Se, Sp and Balanced Accuracy (Bacc) after setting a threshold on the training cohort, number of false negative (FN), positive and negative predictive values (PPV and NPV). Importance of each feature in the final model was also reported. Decision curve analysis (DCA) was also used for inter-model comparison. Full description of the feature set reduction workflow and of the model building is available as Supplementary Protocol 2.

With the same methodology, and given the number of MRI scans used for the acquisition of prostate MRI, an *a posteriori* statistical harmonization method was used. The ComBat harmonization method [24,25] is a statistical method used to remove intersite technical variability while preserving intra-site variability. It was performed before feature set selection and model building creating a ComBat Combined model. For completeness, a model based on clinical/biological features only was also developed.

As a result, three separate models were thus developed and validated: the New-Clinical, the New Combined and the ComBat Combined models.

### 2.5. Inter-Reader Variability

Semi-automatic segmentation was performed by two other experts (U.S. and F.L.) blinded to the results of the previous delineation by V.B in a randomly selected subset (*n* = 18) of the testing set. Protocol regarding analysis of inter-reader variability is described in Supplementary Protocol 3.

We autoevaluated our study based on the radiomics quality score developed by Lambin et al. [26].

### 2.6. Ethics Committee

The study was approved by the hospital ethical committee (NCT04909957). All patients were given 15 days to formulate their opposition.

## 3. Results

### 3.1. Population

From 2010 to 2018, 552 patients underwent radical prostatectomy with eLND, among which 280 patients were finally included with 272 excluded patients due to unavailable or incomplete MRI (*n* = 266) and unanalyzable MRI (*n* = six). The overall population was then randomly divided into a training cohort (*n* = 168) and a testing cohort (*n* = 112). LNI was found in 51 patients (18.2%), equally sampled between the training set (32 patients—19.0%) and the testing set (19 patients—17.0%). The training and testing set were globally comparable, except for the PIRADS classification. Main patients’ characteristics are summarized in Table 1. A flow-chart is proposed as Supplementary Figure S1.

### 3.2. Feature Set Reduction

Regarding clinical features, out of the 11 initial features, 10 were further considered for the model building. For the radiomic features and before statistical harmonization, the feature set reduction allowed a preselection of only four radiomic features: at the first step, out of the initial 8640 features, only five radiomic features with an AUC ≥ 0.70 remained (Supplementary Table S2). They were all from the T2 sequence, four of them from wavelet-filtered images and one shape feature. At the second step (Spearman rank correlation coefficient), all these features showed intra-correlation levels below 0.5 (Supplementary Table S3) except for one, making a total number of four selected radiomic features for model building: Surface Area, Inverse Difference Normalized extracted from the Gray Level Co-Occurrence matrix, Run Entropy extracted from the Gray Level Run Length Matrix, and Contrast extracted from the neighbouring Grey Tone Difference Matrix. Finally, combining all features, the model achieving the highest mean accuracy (New-Combined: 0,97) was based on six features, among which the three most important features were the Inverse Difference Normalized, the percentage of positive biopsy cores and the percentage of positive biopsy cores with highest-grade PCa. With a decremental approach on the mean Bacc, the best combined (New-Combined) model was finally composed of these six features (Supplementary Table S4). With the same methodology but after ComBat harmonization, the ComBat-Combined model consisted in the association of seven features, among which four were radiomic features and the most important feature remaining the percentage of positive biopsy cores with highest-grade PCa. Apart from the Inverse Difference Normalized extracted from the Gray Level Co-Occurence matrix, the three other radiomic features were first order features (Kurtosis, Skewness and Mean). Finally, the clinical model combined five features among which the percentage of positive biopsy cores with highest-grade PCa accounted for 71.0% of the model’s prediction. Composition of each model is available in Table 2.

### 3.3. Training Set

In the training set (*n* = 168), the New-Combined model achieved a high correlation with the risk of LNI (AUC of 1.00, *p* < 0.0001). With a cut-off of 7%, the New-Combined model resulted in a C-Index of 0.98 while with 10%, the C-Index raised to 1.00 (Table 3). These cut-offs allowed almost perfect classification with only six and one patients classified as false positives, for the 7 and 10% cut-offs, respectively (Supplementary Table S5). The New-Clinical and ComBat-Combined models achieved similar high results on the training cohort with an AUC of 1.00 and a balanced accuracy of 100% with their respective thresholds.

By comparison, the Partin, Roach, Yale, MSKCC, Briganti 2012 and 2017 models achieved lower respective C-index values of 0.71, 0.68, 0.56, 0.62, 0.63 and 0.73 (Supplementary Table S5).

The newly developed models had a higher AUC than all other nomograms with an increase of 0.19–0.28, the second highest AUC being achieved by the Briganti 2017 nomogram (Supplementary Table S6).

### 3.4. Testing Set

In the testing set (*n* = 112), the Combat-Combined model was ranked as the most efficient model with a C-Index of 0.89 and a Bacc of 89.4% with the 19% threshold. The New-Combined model achieved the second highest C-indexes: 0.87 for the 7% cut-off and 0.86 for the 10% cut-off; followed by the New-Combined model (0.82). In comparison, the other nomograms with their cut-offs resulted in lower C-indexes of 0.70, 0.65, 0.55, 0.66, 0.64 and 0.73 for Partin’s, Roach’s, Yale’s, MSKCC’s, Briganti’s 2012 and Briganti’s 2017 (Supplementary Table S7).

**Figure 1:**
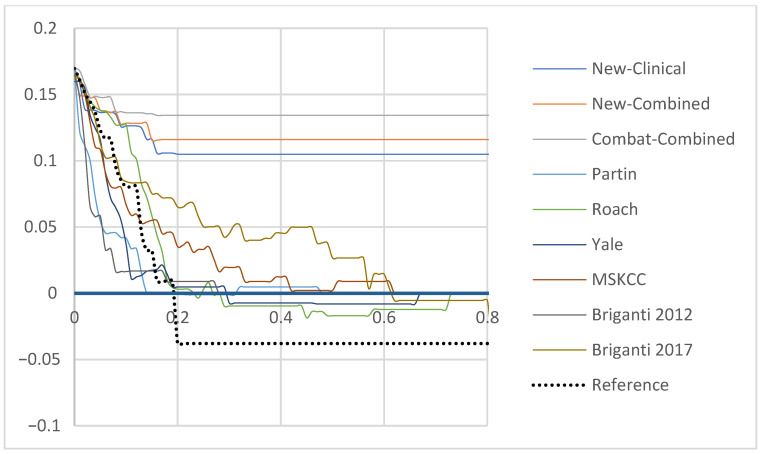
Decision curve analysis demonstrating the net benefit associated with use of the New-Clinical, New-Combined and ComBat-Combined Models for detection of lymph node invasion in comparison to currently available tools in the testing set (Partin, Yale, Roach, Briganti 2012, Briganti 2017 and MSKCC). *x*-axis: predicted probability, *y*-axis: clinical net benefit. Abbreviations: NN: Neural Network, MSKCC: Memorial Sloan Kettering Cancer Center.

The ComBat-Combined model reduced the risk of FN to 3.3% while correctly avoiding the risk of eLND for 88 (96.6%) patients, with the 19% cut-off. Among the 21 patients classified above the 19% cut-off, 76.2%% had a proven LNI resulting in a specificity (Sp) of 89.4%. Detailed results of each model are available in Table S7. Patients classified above the 19% cut-off using the ComBat-Combined model were 23.1 times more likely to present a LNI, respectively (Supplementary Table S8). For comparison, the second best relative-risk ratio, apart from the New-Clinical and New-Combined models, was 8.2 (MSKCC). Finally, DCA revealed that the newly developed models improved LNI risk prediction when compared to other models (Figure 1). Similarly, calibration plots showed a calibration as satisfactory as the Briganti 2017 and the MSKCC nomograms (Supplementary Figure S2).

### 3.5. Inter-Reader Variability

**Figure 2:**
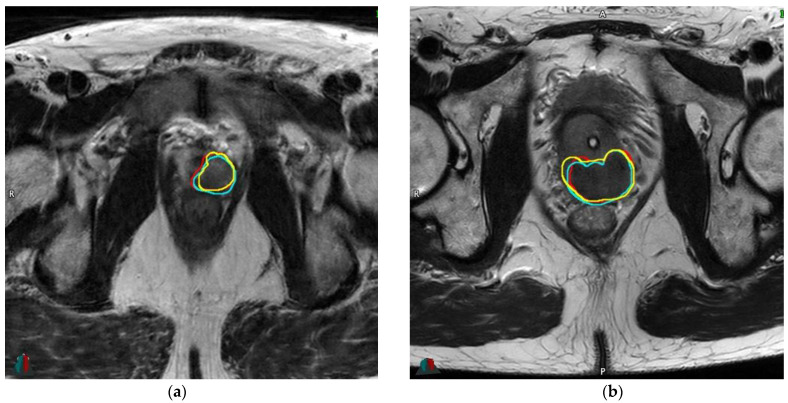
Example of variations of delineations for two patients: (**a**): 56.2 yo, cT2a, PSA 7.7 ng/mL, Gleason 3 + 4 (ISUP 2), respective DICE overlap coefficients with V.B (reference): 0.85 (F.L) and 0.83 (U.S); (**b**): 67.4 yo, cT1c, PSA 1.8 ng/mL, Gleason 4 + 4 (ISUP 4), respective DICE overlap coefficients with V.B (reference): 0.83 (F.L) and 0.81 (U.S). Abbreviations: yo: years old, PSA: prostate-specific antigen, ng: nanograms, mL: millimeter, ISUP: International Society of Urological Pathology. Red: delineation #1 (V.B), cyan: delineation #2 (F.L), yellow: delineation #3 (U.S).

The three independent segmentations achieved a mean Dice overlap coefficient of 0.81 and a mean Hausdorff distance of 0.67 mm (Supplementary Table S9). The ICC between each feature’s value (feature one -> six, respectively) corresponding to the different delineations ranged from 0.85 to 0.98 and from 0.94 to 0.99 for the average measures (Supplementary Table S10). In this subset of patients, three patients had LNI (16.7%). No change in LNI prediction occurred with the changes in radiomic features’ values across the experts’ delineation and whatever the chosen cut-off (Supplementary Table S11). An example of the variations of the delineations for two patients is available as Figure 2.

### 3.6. NCCN Risk Classification

The ComBat-Combined model performed robustly in the intermediate and high-risk patients according to NCCN risk classification with BAccs in the testing set of 86.7 and 94.8%, respectively (Supplementary Table S12).

### 3.7. Subgroup with Targeted Biopsies

Targeted biopsies were performed in a subset of 98 patients (35.0%), with *n* = 62 patients (63.3%) and *n* = 36 patients (36.7%) in the training and testing cohorts, respectively. The Briganti 2018 resulted in AUCs of 0.73 and 0.74 compared to 1.00 and 0.96 for the ComBat-Combined model, in the training and testing cohorts, respectively. When applying the 7% prespecified cut-off, the Briganti 2018 achieved a C-index of 0.68 (Se 75.0%, Sp 60.0%) in the training cohort and a C-index of 0.58 (Se 37.5%, Sp 78.6%) in the testing cohort. The ComBat-Combined model with the 19% cut-off achieved C-Indexes of 1.00 and 0.86 in the training and testing cohorts, respectively. Comparison between the Briganti 2018 and each newly developed model is presented as ROC curves: Supplementary Figure S3a for the training cohort and Supplementary Figure S3b for the testing cohort. The respective DCA and calibration plots for the Briganti 2018, the New-Combined and the ComBat-Combined models are shown as Supplementary Figures S4a–d (training cohort) and S5a–d (testing cohort).

### 3.8. Radiomics Quality Score

Our study scores moderately on the radiomics quality score [26] with 17 points (out of 36, Supplementary Table S13).

## 4. Discussion

According to EAU guidelines [8], eLND completion should be based on the use of predictive tools using disease and patients’ characteristics, such as the models we tested (Briganti 2012 [12], Briganti 2017 [13] and MSKCC [14] nomograms). While these models have shown satisfying results, confirmed with external validation, calibration remained perfectible, especially in intermediate-risk patients. Indeed, in a population of 175 patients with intermediate PCa, the Briganti 2017 nomogram achieved a low AUC of 0.53 [27]. The Briganti 2018, while further enhancing the prediction quality, focuses specifically on patients with software-based fusion targeted biopsies [16]. However, despite being more precise, software-based fusion remains scarce due to logistics consideration, limiting the Briganti’s nomogram’s generalizability, most centers still using cognitive fusion biopsies [28]. Therefore, LNI risk prediction tools and their generalizability need to be improved.

Our Combat-combined model achieved a higher performance when compared to the available models and a higher net benefit according to DCA, even in the testing set. For predicted probabilities ~85–95%, the newly developed models had negative net benefit reflecting the few false negatives that would still be operated on with eLND despite not showing LNI. Application of the ComBat harmonization method enhanced the model’s performance with the Combat-model reaching an Bacc of 89.4% in the testing set, with a threshold set to 19%. We also thoroughly assessed the robustness of our model to delineation’s variations. The LNI risk prediction was not modified by switching the experts’ delineations, despite the slight changes between each segmentation in a subset of 18 randomly selected patients.

Recent models were systematically enhanced by the addition of MRI data but none incorporated radiomics analysis. Radiomic analysis in the PCa setting has been previously studied for both diagnosis and treatment outcome prediction [19,20,29]. LNI risk is known to be correlated to the Gleason score, PSA level and T stage [2]. The better assessment of tumor heterogeneity via radiomic analysis could explain the superiority of such features over simple and independent clinical, biological or radiological data. Among the seven features in the Combat-Combined model, four were radiomics features, accounting for 26.5% of the overall model’s prediction. For instance, the Inverse Difference Normalized (Idn) accounted for 4.6% of the prediction. Idn is a measure of the local homogeneity of an image and normalizes the difference between the neighboring intensity values by dividing over the total number of discrete intensity values: by definition, the higher the value is, the more uniform the volume is and vice versa [30].

The radiomics approach applied to routinely acquired images for diagnosis has the great advantage of being cost-effective and noninvasive. Genomic tests, such as the Decipher Prostate Cancer test^®^, have been used to stratify PCa patients on metastasis-free survival and cancer-specific mortality [31]. Even if costs have been reduced, such genomic tests have never been evaluated for LNI risk stratification.

Our workflow aimed to reduce common statistical biases through a conservative feature set reduction procedure and by relying on separate training and testing sets. However, some limitations persist. First, the use of training and testing sets does not replace a full external validation (which would also enhance the overall radiomics quality score of the study). Previous models were first internally developed using a logistic regression approach and secondly externally validated. To overcome the lack of external validation in our study, we tested our models in an internal set, well separated from the training set. This method showed a high stability of the model between the training and testing sets. We partly addressed that limitation by analyzing a subset of 18 patients with three different experts’ delineations, with no resulting LNI classification changes. The ComBat harmonization method was performed in order to account for the MRI scans’ heterogeneity. It resulted in a higher performing model, the Combat-Combined model. Neural networks are often criticized as being “black boxes” [32]. Our approach offers classification by normalized importance of the features, thus partly addressing this issue and providing models with partial clinical interpretability for the users. Finally, comparison with the latest Briganti 2018 nomogram was only offered in a subset of patients because of the targeted biopsies being nonmandatory in our study. Software-based fusion biopsies are considered the standard of care since 2018 [17] but our set predated the recommendation. However, the benefit of the latest Briganti 2018 over Briganti 2017 and Briganti 2012 appears to be small [33,34].

In patients treated with radiotherapy, pelvic irradiation remains controversial. While some studies suggest a possible benefit for low [35], and high-risk PCa patients [36,37], guidelines for pelvic lymph node irradiation remain weak because of the lack of level 1 data. The lack of consensus underpins the need of better biomarkers.

## 5. Conclusions

Radiomic features extracted from the preoperative MRI seem to provide added predictive value to usual models regarding LNI risk prediction in PCa, in an external testing set. Adoption of this model could avoid up to 80% of eLND with a risk of missing only 1.1% patients with LNI. External and prospective validations are currently under investigation.

## Figures and Tables

**Table 1:** Patient and tumour characteristics in the training and testing sets.

		Training Set		Testing Set		*p*
		*n* = 168 (60.0%)		*n* = 112 (40.0%)		
Age (median, range)		65.0	45.3–79.6	66.7	50.8–77.8	0.10
PSA level (median, range)		7.7	1.5–63.0	7.4	1.0–49.9	0.16
Clinical Tumour Stage (nb, %)	T1c	90	53.6	71	63.4	0.06
	T2a	34	20.2	22	19.6	
	T2b	30	17.9	14	12.5	
	T2c	9	5.	3	2.7	
	T3a	1	0.6	1	0.9	
	T3b	4	2.4	1	0.9	
mpMRI Tumour Stage (nb, %)	T2a	0	0.0	0	0.0	0.60
	T2b	71	42.3	45	40.2	
	T2c	66	39.3	43	38.4	
	T3a	31	18.4	24	21.4	
	≥T3b	0	0	0	0	
PIRADS (nb, %)	3	12	7.1	39	34.8	<0.0001
	4	60	35.7	41	36.6	
	5	96	57.2	32	28.6	
Median maximum index lesion diameter on mpMRI (mm, IQR)		13.2	9.7–16.2	13.6	11.6–15.7	0.26
Number of Lesion PIRADS ≥ 3 on mpMRI per patient (nb, %)	1	153	91.1	100	89.3	0.62
	2	15	8.9	12	10.7	
Type of MRI (nb, %)	Siemens 1.5T	123	73.2	75	67.0	0.26
	Philips 3T	45	26.8	37	33.0	
Biopsy ISUP score (nb, %)	1	38	22.6	19	17.0	0.12
	2	77	45.8	53	47.3	
	3	30	17.9	13	11.6	
	4	23	13.7	23	20.5	
	5	0	0.0	4	3.6	
NCCN risk classification (nb, %)	Low	15	8.9	8	7.1	0.23
	Intermediate	114	67.9	71	63.4	
	High	39	23.2	33	29.5	
Nb Cores (mean, range)		13.7	6–35	13.3	8–24	0.37
Nb Positive Cores (mean, range)		5.9	1–22	5.4	1–12	0.49
Ratio Positive Cores ± (mean, range)		43.5	2.9–100	41.2	5.9–100	0.57
Percentage of positive cores with highest grade (median, range)		30.0	3.2–73.9	29.5	2.6–67.3	0.68
Percentage of positive cores with lower grade (median, range)		28.0	0.5–62.7	27.1	2.2–61.8	0.42
Pathological Tumour Stage (nb, %)	T2a	3	1.8	8	7.1	0.16
	T2b	3	1.8	5	4.5	
	T2c	67	39.9	38	33.9	
	T3a	59	35.1	45	40.2	
	T3b	34	20.2	16	14.3	
	T4	2	1.2	0	0	
Surgical Margin (nb, %)	Positive	88	52.4	66	58.9	0.28
	Negative	80	47.6	46	41.1	
Pathological ISUP score (nb, %)	1	17	10.1	10	8.9	0.23
	2	56	33.3	46	41.1	
	3	50	29.8	36	32.1	
	4	10	6.0	2	1.8	
	5	35	20.8	18	16.1	
Nb Nodes removed (median IQR)		15	12–17	15	12–18	0.44
Nb Nodes positive (median, range)		1	1–2	1	1–2	0.67
Patients with LNI (nb, %)		32	19.0	19	17.0	0.66

Abbreviations: PSA: Prostate Specific Antigen, mpMRI: multiparametric Magnetic Resonance Imaging, nb: number, LNI: Lymph Node Involvement, IQR: InterQuantile, ISUP: Internationl Society of Urological Pathology.

**Table 2:** Composition of the New-Clinical, New-Combined and Combat-Combined Models.

Model	Feature	Importance
New-Clinical Model	PIRADS Score	3.6%
	Percentage of positive biopsy cores with lowest-grade PCa	4.2%
	Clinical Tumour Stage	4.9%
	Percentage of positive biopsy cores	16.3%
	Percentage of positive biopsy cores with highest-grade PCa	71.0%
New-Combined Model	PIRADS Score	3.4%
	Percentage of positive biopsy cores with lower grade Pca	4.0%
	MRI Tumour Stage	4.6%
	Feature 2	6.4%
	Percentage of positive biopsy cores	15.2%
	Percentage of positive biopsy cores with highest-grade PCa	66.4%
Combat-Combined Model	Feature 8	4.6%
	Number of positive biopsy cores	5.7%
	Feature 2	7.0%
	Feature 7	7.2%
	Feature 6	7.7%
	Percentage of positive biopsy cores	12.7%
	Percentage of positive biopsy cores with highest-grade PCa	55.2%

Abbreviations: PCa: Prostate Cancer, MRI = Magnetic Resonnance Imaging, see Supplementary Table S2 for feature’s description.

**Table 3:** Results of the New-Combined model by cut-offs in the training set.

Model Cut-Off	C-Index	Se	Sp	BAcc	Number of Patients, *n* (%)					
					Below the Cutoff (eLND not Recommended)			Above the Cutoff (eLND Recommended)		
					Total	Without LNI	With LNI	Total	Without LNI	With LNI
1%	0.88	100	76.5	88.3	104 (61.9)	104 (100)	0 (0)	64 (38.1)	32 (50.0)	32 (50.0)
2%	0.91	100	81.6	90.8	111 (66.1)	111 (100)	0 (0)	57 (33.9)	25 (43.9)	32 (56.1)
3%	0.93	100	86	93.0	117 (69.6)	117 (100)	0 (0)	51 (30.4)	19 (37.3)	32 (62.7)
4%	0.95	100	89.7	94.9	122 (72.6)	122 (100)	0 (0)	46 (27.4)	14 (30.4)	32 (69.6)
5%	0.96	100	91.9	96.0	125 (74.4)	125 (100)	0 (0)	43 (25.6)	11 (25.6)	32 (74.4)
6%	0.97	100	94.1	97.1	128 (76.2)	128 (100)	0 (0)	40 (23.8)	8 (20.0)	32 (80.0)
7%	0.98	100	95.6	97.8	130 (77.4)	130 (100)	0 (0)	38 (22.6)	6 (15.8)	32 (84.2)
8%	0.98	100	96.3	98.2	131 (78.0)	131 (100)	0 (0)	37 (22.0)	5 (13.5)	32 (86.5)
9%	0.99	100	97.8	98.9	133 (79.2)	133 (100)	0 (0)	35 (20.8)	3 (8.6)	32 (91.4)
10%	1.00	100	99.3	99.7	135 (80.4)	135 (100)	0 (0)	33 (19.6)	1 (3.0)	32 (97.0)
11%	1.00	100	99.3	99.7	135 (80.4)	135 (100)	0 (0)	33 (19.6)	1 (3.0)	32 (97.0)

Abbreviations: eLND: extensive lymph node dissection, LNI: lymph node involvement, Se: Sensitivity, Sp: Specificity, BAcc: Balanced Accuracy.

## Data Availability

All data are presented in the article.

## Supplementary Materials

The following are available online at https://www.mdpi.com/article/10.3390/cancers13225672/s1: Protocol S1: Radiomics features extraction; Protocol S2: Features set reduction and model building; Protocol S3: Inter-reader variability; Figure S1: Flowchart of the patients’ selection; Figure S2: Calibration plots for each available model, the New-Combined model and the Combat-Combined model, in the testing set; Figure S3: Comparison between the Briganti 2018 and the New-Clinical, the New-Combined and the Combat-Combined models ROC (Receiver Operative Characteristics) curves in the training set (3A) and in the testing set (3B); Figure S4: Decision curve analysis (A) and calibration plots for the Briganti 2018 (B), the New-Combined model (C) and the Combat-Combined models (D) in the training set; Figure S5: Decision curve analysis (A) and calibration plots for the Briganti 2018 (B), the New-Combined model (C) and the Combat-Combined models (D) in the testing set; Table S1: Summary of MRI scan acquisition parameters; Table S2: Selected radiomic features after feature selection; Table S3: Spearman’s correlation coefficient (training set); Table S4: Analysis of each built model and respective mean accuracy based on the Boostrap analysis (*n* = 1000 replications) in the training set; Table S5: Analysis of each model’s discrimination between patients with or without LNI in the training set; Table S6: Comparison of ROC curves in the training set; Table S7: Analysis of each model’s discrimination between patients with or without LNI (testing set); Table S8: Relative Risk derived from each model (testing set); Table S9: Inter-reader variability assessment—segmentation; Table S10: Intra-class correlation for each radiomic feature in the New-Combined model, depending on the delineation variation; Table S11: Inter-reader variability assessment—LNI risk prediction; Table S12: Model performance according to the NCCN risk classification; Table S13: Radiomics Quality Score.

## References

[1] EU ECIS: European Cancer Information System. https://ecis.jrc.ec.europa.eu.

[2] Abdollah F., Suardi N., Cozzarini C., Gallina A., Capitanio U., Bianchi M., Sun M., Fossati N., Passoni N.M., Fiorino C. (2012). Selecting the Optimal Candidate for Adjuvant Radiotherapy after Radical Prostatectomy for Prostate Cancer: A Long-term Survival Analysis. Eur. Urol..

[3] Loeb S., Partin A.W., Schaeffer E.M. (2010). Complications of pelvic lymphadenectomy: Do the risks outweigh the benefits?. Rev. Urol..

[4] Briganti A., Chun F., Salonia A., Suardi N., Gallina A., Da Pozzo L.F., Roscigno M., Zanni G., Valiquette L., Rigatti P. (2006). Complications and Other Surgical Outcomes Associated with Extended Pelvic Lymphadenectomy in Men with Localized Prostate Cancer. Eur. Urol..

[5] Mazzone E., Preisser F., Nazzani S., Tian Z., Bandini M., Gandaglia G., Fossati N., Montorsi F., Graefen M., Shariat S. (2018). The Effect of Lymph Node Dissection in Metastatic Prostate Cancer Patients Treated with Radical Prostatectomy: A Contemporary Analysis of Survival and Early Postoperative Outcomes. Eur. Urol. Oncol..

[6] Fossati N., Willemse P.-P.M., Broeck T.V.D., Bergh R.C.V.D., Yuan Y., Briers E., Bellmunt J., Bolla M., Cornford P., De Santis M. (2017). The Benefits and Harms of Different Extents of Lymph Node Dissection during Radical Prostatectomy for Prostate Cancer: A Systematic Review. Eur. Urol..

[7] Touijer K.A., Sjoberg D.D., Benfante N., Laudone V.P., Ehdaie B., Eastham J.A., Scardino P.T., Vickers A. (2021). Limited versus Extended Pelvic Lymph Node Dissection for Prostate Cancer: A Randomized Clinical Trial. Eur. Urol. Oncol..

[8] Mottet N., Cornford P., van den bergh E., Briers E., De Santis M., Fanti S., Gillessen S., Grummet J.P., Henry A.M., Lam T.B. EAU Guidelines: Prostate Cancer 2021. https://uroweb.org/guideline/prostate-cancer/.

[9] Tosoian J.J., Chappidi M., Feng Z., Humphreys E.B., Han M., Pavlovich C.P., Epstein J.I., Partin A.W., Trock B.J. (2016). Prediction of pathological stage based on clinical stage, serum prostate-specific antigen, and biopsy Gleason score: Partin Tables in the contemporary era. BJU Int..

[10] Roach M., Marquez C., Yuo H.-S., Narayan P., Coleman L., Nseyo U.O., Navvab Z., Carroll P.R. (1994). Predicting the risk of lymph node involvement using the pre-treatment prostate specific antigen and gleason score in men with clinically localized prostate cancer. Int. J. Radiat. Oncol..

[11] Yu J.B., Makarov D., Gross C. (2011). A New Formula for Prostate Cancer Lymph Node Risk. Int. J. Radiat. Oncol..

[12] Briganti A., Larcher A., Abdollah F., Capitanio U., Gallina A., Suardi N., Bianchi M., Sun M., Freschi M., Salonia A. (2011). Updated Nomogram Predicting Lymph Node Invasion in Patients with Prostate Cancer Undergoing Extended Pelvic Lymph Node Dissection: The Essential Importance of Percentage of Positive Cores. Eur. Urol..

[13] Gandaglia G., Fossati N., Zaffuto E., Bandini M., Dell’Oglio P., Bravi C.A., Fallara G., Pellegrino F., Nocera L., Karakiewicz P.I. (2017). Development and Internal Validation of a Novel Model to Identify the Candidates for Extended Pelvic Lymph Node Dissection in Prostate Cancer. Eur. Urol..

[14] Godoy G., Chong K.T., Cronin A., Vickers A., Laudone V., Touijer K., Guillonneau B., Eastham J.A., Scardino P.T., Coleman J. (2011). Extent of Pelvic Lymph Node Dissection and the Impact of Standard Template Dissection on Nomogram Prediction of Lymph Node Involvement. Eur. Urol..

[15] Ahdoot M., Wilbur A.R., Reese S.E., Lebastchi A.H., Mehralivand S., Gomella P., Bloom J., Gurram S., Siddiqui M., Pinsky P. (2020). MRI-Targeted, Systematic, and Combined Biopsy for Prostate Cancer Diagnosis. N. Engl. J. Med..

[16] Gandaglia G., Ploussard G., Valerio M., Mattei A., Fiori C., Fossati N., Stabile A., Beauval J.-B., Malavaud B., Roumiguié M. (2018). A Novel Nomogram to Identify Candidates for Extended Pelvic Lymph Node Dissection among Patients with Clinically Localized Prostate Cancer Diagnosed with Magnetic Resonance Imaging-targeted and Systematic Biopsies. Eur. Urol..

[17] Kasivisvanathan V., Rannikko A., Borghi M., Panebianco V., Mynderse L.A., Vaarala M., Briganti A., Budäus L., Hellawell G., Hindley R.G. (2018). MRI-Targeted or Standard Biopsy for Prostate-Cancer Diagnosis. N. Engl. J. Med..

[18] Meng X., Rosenkrantz A., Huang R., Deng F.-M., Wysock J.S., Bjurlin M.A., Huang W., Lepor H., Taneja S.S. (2018). The Institutional Learning Curve of Magnetic Resonance Imaging-Ultrasound Fusion Targeted Prostate Biopsy: Temporal Improvements in Cancer Detection in 4 Years. J. Urol..

[19] Bourbonne V., Fournier G., Vallières M., Lucia F., Doucet L., Tissot V., Cuvelier G., Hue S., Du H.L.P., Perdriel L. (2020). External Validation of an MRI-Derived Radiomics Model to Predict Biochemical Recurrence after Surgery for High-Risk Prostate Cancer. Cancers.

[20] Bourbonne V., Vallières M., Lucia F., Doucet L., Visvikis D., Tissot V., Pradier O., Hatt M., Schick U. (2019). MRI-Derived Radiomics to Guide Post-operative Management for High-Risk Prostate Cancer. Front. Oncol..

[21] Yasaka K., Akai H., Kunimatsu A., Kiryu S., Abe O. (2018). Deep learning with convolutional neural network in radiology. Jpn. J. Radiol..

[22] Heidenreich A., Ohlmann C.H., Polyakov S. (2007). Anatomical Extent of Pelvic Lymphadenectomy in Patients Undergoing Radical Prostatectomy. Eur. Urol..

[23] Turkbey B., Rosenkrantz A.B., Haider M.A., Padhani A., Villeirs G., Macura K.J., Tempany C.M., Choyke P.L., Cornud F., Margolis D.J. (2019). Prostate Imaging Reporting and Data System Version 2.1: 2019 Update of Prostate Imaging Reporting and Data System Version 2. Eur. Urol..

[24] Fortin J.-P., Cullen N., Sheline Y.I., Taylor W.D., Aselcioglu I., Cook P.A., Adams P., Cooper C., Fava M., McGrath P.J. (2017). Harmonization of cortical thickness measurements across scanners and sites. NeuroImage.

[25] Johnson W., Li C., Rabinovic A. (2006). Adjusting batch effects in microarray expression data using empirical Bayes methods. Biostatistics.

[26] Lambin P., Leijenaar R.T., Deist T.M., Peerlings J., De Jong E.E., van Timmeren J., Sanduleanu S., LaRue R.T., Even A.J., Jochems A. (2017). Radiomics: The bridge between medical imaging and personalized medicine. Nat. Rev. Clin. Oncol..

[27] Peilleron N., Seigneurin A., Herault C., Verry C., Bolla M., Rambeaud J.-J., Descotes J.-L., Long J.-A., Fiard G. (2020). External evaluation of the Briganti nomogram to predict lymph node metastases in intermediate-risk prostate cancer patients. World J. Urol..

[28] Cornud F., Roumiguié M., De Longchamps N.B., Ploussard G., Bruguière E., Portalez D., Malavaud B. (2018). Precision Matters in MR Imaging–targeted Prostate Biopsies: Evidence from a Prospective Study of Cognitive and Elastic Fusion Registration Transrectal Biopsies. Radiology.

[29] Wibmer A., Hricak H., Gondo T., Matsumoto K., Veeraraghavan H., Fehr D., Zheng J., Goldman D., Moskowitz C., Fine S.W. (2015). Haralick texture analysis of prostate MRI: Utility for differentiating non-cancerous prostate from prostate cancer and differentiating prostate cancers with different Gleason scores. Eur. Radiol..

[30] Zwanenburg A., Vallières M., Abdalah M.A., Aerts H.J.W.L., Andrearczyk V., Apte A., Ashrafinia S., Bakas S., Beukinga R.J., Boellaard R. (2020). The Image Biomarker Standardization Initiative: Standardized Quantitative Radiomics for High-Throughput Image-based Phenotyping. Radiology.

[31] Dalela D., Loppenberg B., Sood A., Sammon J., Abdollah F. (2016). Contemporary Role of the Decipher(R) Test in Prostate Cancer Management: Current Practice and Future Perspectives. Rev. Urol..

[32] Clark T., Nyberg E. (2020). Creating the Black Box: A Primer on Convolutional Neural Network Use in Image Interpretation. Curr. Probl. Diagn. Radiol..

[33] Gandaglia G., Martini A., Ploussard G., Fossati N., Stabile A., De Visschere P., Borgmann H., Heidegger I., Steinkohl F., Kretschmer A. (2020). External Validation of the 2019 Briganti Nomogram for the Identification of Prostate Cancer Patients Who Should Be Considered for an Extended Pelvic Lymph Node Dissection. Eur. Urol..

[34] Diamand R., Oderda M., Albisinni S., Fourcade A., Fournier G., Benamran D., Iselin C., Fiard G., Descotes J.-L., Assenmacher G. (2020). External validation of the Briganti nomogram predicting lymph node invasion in patients with intermediate and high-risk prostate cancer diagnosed with magnetic resonance imaging-targeted and systematic biopsies: A European multicenter study. Urol. Oncol. Semin. Orig. Investig..

[35] Pommier P., Chabaud S., Lagrange J.-L., Richaud P., Le Prise E., Wagner J.-P., Azria D., Beckendorf V., Suchaud J.-P., Bernier V. (2016). Is There a Role for Pelvic Irradiation in Localized Prostate Adenocarcinoma? Update of the Long-Term Survival Results of the GETUG-01 Randomized Study. Int. J. Radiat. Oncol..

[36] Sandler K.A., Cook R., Ciezki J.P., Ross A.E., Pomerantz M.M., Nguyen P.L., Shaikh T., Tran P.T., Stock R.G., Merrick G.S. (2019). Prostate-only Versus Whole-pelvis Radiation with or without a Brachytherapy Boost for Gleason Grade Group 5 Prostate Cancer: A Retrospective Analysis. Eur. Urol..

[37] Murthy V., Maitre P., Kannan S., Panigrahi G., Krishnatry R., Bakshi G., Prakash G., Pal M., Menon S., Phurailatpam R. (2021). Prostate-Only versus Whole-Pelvic Radiation Therapy in High-Risk and Very High-Risk Prostate Cancer (POP-RT): Outcomes from Phase III Randomized Controlled Trial. J. Clin. Oncol..

