# Supplementary material for: Development of a Radiomic-Based Model Predicting Lymph Node Involvement in Prostate Cancer Patients
Source: Cancers (Basel). 2021 Nov 12;13(22):5672. doi: 10.3390/cancers13225672 (PMC8616049; doi:10.3390/cancers13225672)
Supplement: Supplementary file 1 [file cancers-13-05672-s001.zip › cancers-1413572-supplementary.pdf]

SUPPLEMENTARY MATERIAL

PROTOCOL

Supplementary Protocol 1: Radiomics features extraction

Supplementary Protocol 2: Features set reduction and model building

Supplementary Protocol 3: Inter-reader variability

TABLES

Supplementary Table 1 : Summary of MRI scan acquisition parameters

Supplementary Table 2: Selected radiomic features after feature selection

Supplementary Table 3: Spearman's correlation coefficient (training set)

Supplementary Table 4: Analysis of each built model and respective mean accuracy based on the Boostrap analysis (*n = 1000* replications) in the training set

Supplementary Table 5: Analysis of each model's discrimination between patients with or without LNI in the training set

Supplementary Table 6: Comparison of ROC curves in the training set

Supplementary Table 7: Analysis of each model's discrimination between patients with or without LNI (testing set)

Supplementary Table 8: Relative Risk derived from each model (testing set)

Supplementary Table 9: Inter-reader variability assessment – segmentation

Supplementary Table 10: Intra-class correlation for each radiomic feature depending on the delineation variation

Supplementary Table 11: Inter-reader variability assessment – LNI risk prediction

Supplementary Table 12: Model performance according to the NCCN risk classification

Supplementary Table 13: Radiomics Quality Score

FIGURES

Supplementary Figure 1: Flowchart of the patients' selection

Supplementary Figure 2: Calibration plots for each available model, the New-Combined model and the ComBat-Combined model, in the testing set.

Supplementary Figure 3: Comparison between the Briganti 2018 and the New-Clinical, the New-Combined and the ComBat-Combined models ROC (*Receiver Operative Characteristics)* curves in the training set (3A) and in the testing set (3B)

Supplementary Figure 4: Decision curve analysis (A) and calibration plots for the Briganti 2018 (B), the New-Combined model (C) and the ComBat-Combined models (D) in the training set

Supplementary Figure 5: Decision curve analysis (A) and calibration plots for the Briganti 2018 (B), the New-Combined model (C) and the ComBat-Combined models (D) in the testing set

Supplementary Protocol 1: Radiomics features extraction

The index lesion was semi-automatically delineated by a single expert (V.B) using the Fast GrowCut tool imbedded in SlicerRT©. Before feature extraction, a multiscale wavelet decomposition was applied onto the image, resulting in 9 different images for each sequence (original + 8 wavelet subbands)[24]. We then extracted a set of 120 radiomic features (shape-based, first-order statistics and texture features with a fixed bin value of 8, 16, 32 and 64 bins), using the PyRadiomics module incorporated in SlicerRT© on both the apparent diffusion coefficient (ADC) corrected map and the T2 sequence, in accordance with the IBSI (Image Biomarker Standardization Initiative) guidelines[25]. As a result, 8651 features (11 clinical and 120 × 4 (fixed bin value) × 9 (original + 8 wavelet filters) × 2 (MRI sequences) = 8640 radiomic features) were available for each patient.

Supplementary Protocol 2: Features set reduction and model building

The model was built on the training set alone consisting of approximately 60% of the overall cohort. It was then applied on the rest of the patients, defining the testing set (40%).

Regarding the clinical features, when two features exhibited a Spearman rank correlation coefficient (ρ) > 0.7, only the feature with the highest area under the Receiver Operative Characteristic (ROC) curve (AUC) was kept. Regarding the radiomic features and in order to reduce the high number of radiomic features and avoid overfitting, a strict feature set reduction workflow was developed: Correlation between each feature and outcome (presence/absence of LNI) was evaluated in a univariate way using the ROC AUC. Only features with a high correlation (AUC ≥ 0.70) were further considered. Following this first step, features were further selected based on their level of intracorrelation. When two features exhibited a Spearman rank correlation coefficient (ρ) > 0.7, only the feature with the highest AUC was kept. All pre-selected clinical and radiomic features were then considered for the New-Combined model building.

Features were entered as input into a NN approach (Multilayer Perceptron Network, SPSS Modeler v24.0©) with the Boostrap Aggregating tool to improve stability and robustness. The bootstrap method uses randomized sampling with replacement to create *n* (here we used *n = 1000)* different samples. The last step consisted in decrementally reducing the number of retained features in the model by deleting the least important predictor and retraining the model. For each retrained model, the mean accuracy was calculated for the *n = 1000* replications, the model achieving the highest mean accuracy being the selected model and further defined as the New-Combined model.

Several cut-offs were tested for their discrimination between patients at high and low risks of LNI. Evaluation of the trained model was carried out using the AUC along with the C-Index, Se, Sp and Balanced Accuracy (Bacc), number of false negative (FN), positive and negative predictive values (PPV and NPV). Importance of each feature in the final model was also reported.

The same trained model was then evaluated on the remaining patients (testing set) who were not used before.

AUC (in the training set alone), C-Index, Se, Sp, NPV, PPV and Bacc were also used to evaluate the model and to compare it to the available models (Partin[9], Yale[11], Roach[10], Briganti 2012[12], Briganti 2017[13] and MSKCC[14]). ROC curves from each model were compared to the newly developed model[26].

A sub-analysis according to the NCCN risk classification was also performed. As targeted biopsies were not available for all patients, the Briganti 2018 model was only tested in the subgroup of patients who had targeted biopsies. All nomograms were calculated using the Model Validation provided by www.evidencio.com, a free-access web tool which was also used for providing the calibration plots. Decision curves for the overall cohort, training and testing sets were drawn plotting the net benefit with its corresponding threshold.

Supplementary Protocol 3: Inter-reader variability

Variations between each delineation were evaluated using the average Hausdorff distance and the Dice coefficient[27]. Variations of each selected radiomic feature were quantified and intraclass correlation coefficients (ICC) were used to evaluate their agreement across different delineations. Additionally, the resulting impact of the delineation variations on the classification (LNI risk) of the patients was reported.

Supplementary Table 1 : Summary of MRI scan acquisition parameters

**Acquisition parameters**	**Siemens 1.5T**	**Philips Achieva 3T**
Number of patients	75	32
Magnetic field strength (Tesla)	1.5T	3T
T2-Weighted		
Matrix (pixels)	192 × 192	268 × 268
Field of view (mm)	250 × 250	320 × 320
ET (ms)	110	90
RT (ms)	2500	4500
Slice Thickness (mm)	1.5	1.5
ADC map		
Matrix (pixels)	128 × 128	144 × 144
Field of view (mm)	200 × 200	240 × 240
ET (ms)	80	80
RT (ms)	2300	2300
Slice Thickness (mm)	3.5	3.5
Diffusion gradient	B50-400-1000	B100-600-1000

Anatomical images (axial turbo spin echo T2-weighted) were combined with functional sequences, such as axial diffusion sequences using several b-values up to 1100 and dynamic contrast-enhanced sequences (perfusion sequence for Philips 3T and a T1 sequence with gadolinium injection for Siemens 1.5T). Dynamic contrast-enhanced sequences weren't mandatory for inclusion. ADC maps were calculated using each corresponding manufacturer's software.

Abbreviations: RT: repetition time, ET: echo time

Supplementary Table 2: Selected radiomic features before feature set reduction

ComBat/NonComBat	Feature	Volume of Interest	Image sequence	Image Type	Feature Class	Feature Name	Bin
Non ComBat	Feature 1	Lesion	T2	original	shape	Surface Area	8
	Feature 2	Lesion	T2	wavelet-HLL	glcm	Idn	8
	Feature 3	Lesion	T2	wavelet-HHH	glrlm	Run Entropy	64
	Feature 4	Lesion	T2	wavelet-HLL	glszm	Large Area High Gray Level Emphasis	64
	Feature 5	Lesion	T2	wavelet-LLL	ngtdm	Contrast	64
ComBat	Feature 6	Lesion	T2	wavelet-LLL	First order	Kurtosis	8
	Feature 7	Lesion	T2	Original	First order	Skewness	8
	Feature 8	Lesion	T2	wavelet-LLH	First order	Mean	8

Abbreviations: Wavelet (HLL ; High Low Low, HHH : High High High, LLL : Low Low Low), Matrix (glcm : grey-level co-occurrence matrix, glrlm : grey level run-length matrix, ngtdm : neigbouring grey tone difference matrix, glszm : grey-level size zone matrix), Idn: Inverse Difference Normalized

Supplementary Table 3: Spearman's correlation coefficient (training set)

Correlation	Feature 1	Feature 2	Feature 3	Feature 4	Feature 5	Feature 6	Feature 7	Feature 8
Feature 1	1	0.18*	0.04	0.29*	-0.02	-	-	-
Feature 2	0.18*	1	-0.18*	0.87*	-0.44*	0.52*	0.49*	0.12
Feature 3	0.04	-0.18*	1	-0.27*	0.19*	-	-	-
Feature 4	0.29*	0.87*	-0.27*	1	-0.67*	-	-	-
Feature 5	-0.02	-0.44*	0.19*	-0.67*	1	-	-	-
Feature 6	-	0.52*	-	-	-	1	0.19	0.67*
Feature 7	-	0.49*	-	-	-	0.19	1	0.12
Feature 8	-	0.12	-	-	-	0.67*	0.12	1

*Abbreviations: *: $p < 0.01$, see Supplementary Table 2 for feature's description*

Supplementary Table 4: Analysis of each built model and respective mean accuracy based on the Boostrap analysis (*n = 1000* replications) in the training set

Nb of features	New-Clinical	New-Combined	ComBat-Combined
7	-	-	0.97
6	-	0.97	0.96
5	0.98	0.96	0.96
4	0.96	0.96	0.96
3	0.97	0.95	0.95
2	0.95	0.95	0.95
1	0.94	0.94	0.94

Abbreviations: Nb: number

Supplementary Table 5: Analysis of each model's discrimination between patients with or without LNI in the training set

Model	Model cut-off	C-index	Se	Sp	BAcc	Number of patients, n (%)					
						Below the cutoff (eLND not recommended)			Above the cutoff (eLND recommended)		
						Total	Without LNI	With LNI	Total	Without LNI	With LNI
Partin	2%	0.71	78.1	63.2	70.7	93 (55.4)	86 (92.5)	7 (7.5)	76 (44.6)	50 (66.7)	25 (33.3)
Roach	15%	0.68	75.0	61.8	68.4	92 (54.8)	84 (91.3)	8 (8.7)	76 (45.2)	52 (68.4)	24 (31.6)
Yale	15%	0.56	21.9	90.4	56.2	148 (88.1)	123 (83.1)	25 (16.9)	20 (11.9)	13 (65.0)	7 (35.0)
MSKCC	2%	0.62	96.9	27.9	62.4	39 (23.2)	38 (97.4)	1 (2.6)	129 (76.8)	98 (76.0)	31 (24.0)
Briganti 2012	5%	0.63	40.6	85.3	63.0	135 (80.4)	116 (85.9)	19 (14.1)	33 (19.6)	20 (60.6)	13 (39.4)
Briganti 2017	7%	0.73	78.1	67.7	72.9	99 (58.9)	92 (92.9)	7 (7.1)	69 (41.1)	44 (63.8)	25 (36.2)
New-Clinical	26%	1.00	100.0	100.0	100.0	136 (80.95)	136 (100)	0 (0.0)	32 (19.05)	0 (0.0)	32 (100.0)
New-Combined	7%	0.98	100	95.6	97.8	130 (77.4)	130 (100)	0 (0)	38 (22.6)	6 (15.8)	32 (84.2)
New-Combined	10%	1.00	100	99.3	99.7	135 (80.4)	135 (100)	0 (0)	33 (19.6)	1 (3.0)	32 (97.0)
ComBat-Combined	19%	1.00	100.0	100.0	100.0	136 (80.95)	136 (100)	0 (0.0)	32 (19.05)	0 (0.0)	32 (100.0)

Abbreviations: eLND : extensive lymph node dissection, LNI: lymph node involvement, MSKCC: Memorial Sloan Kettering Cancer Center, Se: Sensitivity, Sp: Specificity, BAcc: Balanced Accuracy

Supplementary Table 6: Comparison of ROC curves in the training set

	New-Clinical		New-Combined		ComBat-Model	
Tested Model	Difference between AUC	*p*	Difference between AUC	*p*	Difference between AUC	*p*
Partin	0.27	< 0.0001	0.27	*< 0,0001*	*0.04*	< 0.0001
Roach	0.28	< 0.0001	0.28	*< 0,0001*	*0.04*	< 0.0001
Yale	0.27	< 0.0001	0.27	*< 0,0001*	*0.04*	< 0.0001
MSKCC	0.22	< 0.0001	0.22	*< 0,0001*	*0.04*	< 0.0001
Briganti 2012	0.24	< 0.0001	0.24	*< 0,0001*	*0.04*	< 0.0001
Briganti 2017	0.04	< 0.0001	0.19	*< 0,0001*	*0.04*	< 0.0001
New-Clinical	-	-	0.00	-	*0.00*	-
New-Combined	0.00	-	-	-	*0.00*	-
ComBat-Combined	0.00	-	0.00	-	-	-

Abbreviations: AUC: Area Under the Curve, MSKCC: Memorial Sloan Kettering Cancer Center

Supplementary Table 7: Analysis of each model's discrimination between patients with or without LNI in the testing set

Model	Model cut-off	C-index	Se	Sp	BAcc	Number of patients, n (%)					
						Below the cutoff (eLND not recommended)			Above the cutoff (eLND recommended)		
						Total	Without LNI	With LNI	Total	Without LNI	With LNI
Partin	2%	0.70	68.4	72.0	70.2	73 (65.2)	67 (91.8)	6 (8.2)	39 (34.8)	26 (66.7)	13 (33.3)
Roach	15%	0.65	63.2	66.7	65.0	69 (61.6)	62 (89.9)	7 (10.1)	43 (38.4)	31 (72.1)	12 (17.9)
Yale	15%	0.55	15.8	93.6	54.7	103 (92.0)	87 (84.5)	16 (15.5)	9 (8.0)	6 (66.7)	3 (33.3)
MSKCC	2%	0.66	94.7	36.6	65.7	35 (31.2)	34 (97.1)	1 (2.9)	77 (68.7)	59 (76.6)	18 (23.4)
Briganti 2012	5%	0.64	36.8	90.3	63.6	96 (85.7)	84 (87.5)	12 (12.5)	16 (14.3)	9 (56.2)	7 (43.8)
Briganti 2017	7%	0.73	73.7	72.0	72.9	76 (67.9)	70 (92.1)	6 (7.9)	36 (32.1)	23 (63.9)	13 (32.1)
New-Clinical	26%	0.82	68.4	94.6	81.5	94 (83.9)	88 (93.6)	6 (6.4)	18 (16.1)	5 (27.8)	13 (72.2)

New-Combined	7%	0.87	84.2	89.3	86.8	86 (76.8)	83 (96.5)	3 (3.5)	26 (23.2)	10 (38.5)	16 (61.5)
New-Combined	10%	0.86	79.0	93.6	86.3	91 (81.2)	87 (95.6)	4 (4.4)	21 (18.8)	6 (28.6)	15 (71.4)
ComBat-Combined	19%	0.89	84.2	94.6	89.4	91 (81.2)	88 (96.6)	3 (3.3)	21 (18.8)	5 (23.8)	16 (76.2)

Abbreviations: eLND : extensive lymph node dissection, LNI: lymph node involvement, MSKCC: Memorial Sloan Kettering Cancer Center, Se: Sensitivity, Sp: Specificity, BAcc: Balanced Accuracy

Supplementary Table 8: Relative Risk derived from each model (testing set)

Model	Cut-off	Relative Risk	*p*
Partin	2%	4.1	*0.002*
Roach	15%	2.8	*0.02*
Yale	15%	2.2	*0.15*
MSKCC	7%	8.2	*0.04*
Briganti 2012	2%	3.5	*0.001*
Briganti 2017	5%	4.6	*0.0007*
New-Clinical	26%	11.3	*< 0.0001*
New-Combined	7%	7.8	*< 0.0001*
New-Combined	10%	12.2	*< 0.0001*
ComBat-Combined	19%	23.1	*< 0.0001*

Abbreviations: MSKCC: Memorial Sloan Kettering Cancer Center

Supplementary Table 9: Inter-reader variability assessment - segmentation

PatientID	Hausdorff E1-E2 (mm)	Dice E1 - E2	Hausdorff E1 - E3 (mm)	Dice E1 - E3	Hausdorff E2 - E3 (mm)	Dice E2 - E3
Patient#001	0.42	0.83	0.43	0.84	0.35	0.88
Patient#002	1.26	0.73	0.47	0.80	0.91	0.82
Patient#003	1.02	0.74	1.05	0.75	0.67	0.81
Patient#004	0.84	0.80	0.82	0.79	0.99	0.80
Patient#005	0.50	0.80	1.36	0.69	1.00	0.79
Patient#006	1.25	0.78	1.34	0.77	0.40	0.90
Patient#007	0.39	0.83	0.48	0.80	0.23	0.89
Patient#008	0.53	0.81	0.51	0.80	0.16	0.90
Patient#009	0.94	0.76	1.21	0.72	0.70	0.86
Patient#010	0.57	0.82	0.88	0.79	0.37	0.89
Patient#011	0.47	0.80	0.42	0.83	0.26	0.86
Patient#012	0.18	0.84	0.13	0.87	0.07	0.92
Patient#013	1.15	0.69	0.30	0.81	0.87	0.79
Patient#014	0.28	0.86	0.78	0.70	0.63	0.76
Patient#015	0.72	0.80	0.89	0.77	0.29	0.89
Patient#016	0.82	0.77	0.62	0.82	0.53	0.82
Patient#017	0.68	0.85	0.75	0.83	0.51	0.87
Patient#018	0.96	0.83	1.19	0.81	0.76	0.87

Abbreviations: E1-3: Expert 1-3 (1: VB, 2 : FL, 3 : US)

Supplementary Table 10: Intra-class correlation for each radiomic feature depending on the delineation variation

Feature	Single Measure		Average Measures
	ICC	CI95%	ICC
Feature 1	0.96	0.90-0.98	0.99
Feature 2	0.85	0.70-0.93	0.94
Feature 3	0.98	0.96-0.99	0.99
Feature 5	0.96	0.91-0.98	0.99
Feature 7	0.97	0.94-0.98	0.98
Feature 8	0.90	0.87-0.92	0.93
Feature 9	0.93	0.90-0.95	0.94

Abbreviations: ICC: Intra-Class Correlation, CI: Confidence Interval, for feature definition: see Supplementary Table 2

Supplementary Table 11: Inter-reader variability assessment – LNI risk prediction with the ComBat-Combined Model

PatientID	Age	PSA	Gleason score	Clinical T stage	MRI T stage	ProbVB	ClassVB	ProbFL	ClassFL	ProbUS	ClassUS	Ground Truth
Patient#001	65.95	12.40	3 + 4	T2b	T2c	0	0	0	0	0	0	0
Patient#002	56.22	7.70	3 + 4	T2b	T3a	0	0	0	0	0	0	0
Patient#003	67.41	1.83	4 + 4	T2a	T3a	0.9	0	1.0	0	0.8	0	0
Patient#004	72.13	26.55	4 + 5	T2c	T3a	0	1	0	1	0	1	0
Patient#005	54.65	4.49	3 + 3	T2b	T2a	0.1	0	0.4	0	0.1	0	0
Patient#006	68.38	7.35	3 + 4	T1c	T2c	0	1	0	1	0	1	0
Patient#007	72.53	14.06	3 + 4	T1c	T2b	0	0	0	0	0	0	0
Patient#008	64.95	4.90	3 + 3	T1c	T2c	0	0	0	0	0	0	0
Patient#009	70.91	7.65	4 + 3	T2a	T3a	0	0	0	0	0	0	0
Patient#010	66.88	7.50	5 + 4	T1c	T2c	0.5	0	0.5	0	0.3	0	0
Patient#011	61.19	6.60	3 + 3	T1c	T2b	1.5	0	1.0	0	0.9	0	0
Patient#012	73.16	10.00	4 + 3	T2a	T2c	0	0	0	0	0	0	0
Patient#013	70.5	6.09	3 + 3	T1c	T2b	0	0	0	0	0	0	0
Patient#014	53.52	4.20	3 + 3	T1c	T2b	0	0	0	0	0	0	0
Patient#015	65.43	14.00	3 + 3	T2a	T2a	2	0	3	0	2	0	0
Patient#016	71.27	7.50	3 + 4	T1c	T2c	0	0	0	0	0	0	1
Patient#017	62.66	7.99	4 + 3	T1c	T2b	98.4	1	98.2	1	97.7	1	1
Patient#018	62.54	9.40	3 + 4	T1c	T2c	99.8	1	99.2	1	99.4	1	1

Abbreviations: PSA : Prostate Specific Antigen, Clinical T stage : clinical tumour stage, MRI T stage : tumour stage based on the Magnetic Resonance Imaging, Prob : Calculated probability (%) of LNI with the novel model and the selected delineation (VB, FL or US). Class : Predicted class (0 : no LNI, 1 : LNI) depending on the calculated probability and the 7% cut-off

Supplementary Table 12: ComBat-Combined model performance according to the NCCN risk classification

NCCN risk	Training set (n = 168)				Testing set (n = 112)			
	Nb	AUC	*p*	Bacc	Nb	AUC	*p*	Bacc
**Low**	15	1.00	**< *0.0001***	100.0	8	1.00	**< *0.0001***	100.0
**Intermediate**	114	1.00	**< *0.0001***	100.0	71	0.97	**< *0.0001***	86.7
**High**	39	1.00	**< *0.0001***	100.0	33	0.98	**< *0.0001***	94.8

Abbreviations: Nb : number of patients, AUC : Area Under the Curve, Bacc : Balanced Accuracy

Supplementary Table 13: Radiomics Quality Score

Item	Points	Current Study
Image protocol quality – well-documented image protocols (e.g.. contrast. slice thickness. energy. etc.) and/or usage of public image protocols allow reproducibility/replicability	+1 (if protocols are well-documented)+1 (if public protocol is used)	2
Multiple segmentations – possible actions are: segmentation by different physicians/algorithms/software. perturbing segmentations by (random) noise. segmentation at different breathing cycles. Analyze feature robustness to segmentation variabilities	+1	1
Phantom study on all scanners – detect inter-scanner differences and vendor-dependent features. Analyze feature robustness to these sources of variability	+1	0
Imaging at multiple time points – collect individuals' images at additional time points. Analyze feature robustness to temporal variabilities (e.g.. organ movement. organ expansion/shrinkage).	+1	0
Feature reduction or adjustment for multiple testing – decreases the risk of overfitting. Overfitting is inevitable if the number of features exceeds the number of samples. Consider feature robustness when selecting features	−3 (if neither measure is implemented)+3 (if either measure is implemented)	3
Multivariable analysis with non radiomic features (e.g.. EGFR mutation) – is expected to provide a more holistic model. Permits correlating/inferencing between radiomics and non radiomics features	+1	1
Detect and discuss biological correlates – demonstration of phenotypic differences (possibly associated with underlying gene–protein expression patterns) deepens understanding of radiomics and biology	+1	0
Cut-off analyses – determine risk groups by either the median. a previously published cut-off or report a continuous risk variable. Reduces the risk of reporting overly optimistic results	+1	1
Discrimination statistics – report discrimination statistics (e.g.. C-statistic. ROC curve. AUC) and their statistical	+1 (if a discrimination statistic and its statistical significance are reported)+1 (if	1

significance (e.g.. p-values. confidence intervals). One can also apply resampling method (e.g.. bootstrapping. cross-validation)	also an resampling method technique is applied)	
Calibration statistics – report calibration statistics (e.g.. Calibration-in-the-large/slope. calibration plots) and their statistical significance (e.g.. p-values. confidence intervals). One can also apply resampling method (e.g.. bootstrapping. cross-validation)	+1 (if a calibration statistic and its statistical significance are reported)+1 (if also an resampling method technique is applied)	1
Prospective study registered in a trial database – provides the highest level of evidence supporting the clinical validity and usefulness of the radiomics biomarker	+7 (for prospective validation of a radiomics signature in an appropriate trial)	0
Comparison to 'gold standard' – assess the extent to which the model agrees with/is superior to the current 'gold standard' method (e.g.. TNM-staging for survival prediction). This comparison shows the added value of radiomics	−5 (if validation is missing)+2 (if validation is based on a dataset from the same institute)+3 (if validation is based on a dataset from another institute)+4 (if validation is based on two datasets from two distinct institutes)+4 (if the study validates a previously published signature)+5 (if validation is based on three or more datasets from distinct institutes)*Datasets should be of comparable size and should have at least 10 events per model feature.	2
Potential clinical utility – report on the current and potential application of the model in a clinical setting (e.g.. decision curve analysis)	+2	2
Cost-effectiveness analysis – report on the cost-effectiveness of the clinical application (e.g.. quality adjusted life years generated)	+2	2
Open science and data – make code and data publicly available. Open science facilitates knowledge transfer and reproducibility of the study	+1	0
Comparison to 'gold standard' – assess the extent to which the model agrees with/is superior to the current 'gold standard' method (e.g.. TNM-staging for survival prediction). This comparison shows the added value of radiomics	+1 (if scans are open source)+1 (if region of interest segmentations are open source)+1 (if code is open source)+1 (if radiomics features are calculated on a set of representative ROIs and the calculated features + representative ROIs are open source)	1
Total	36	17

Supplementary Figure 1: Flowchart of the patients' selection

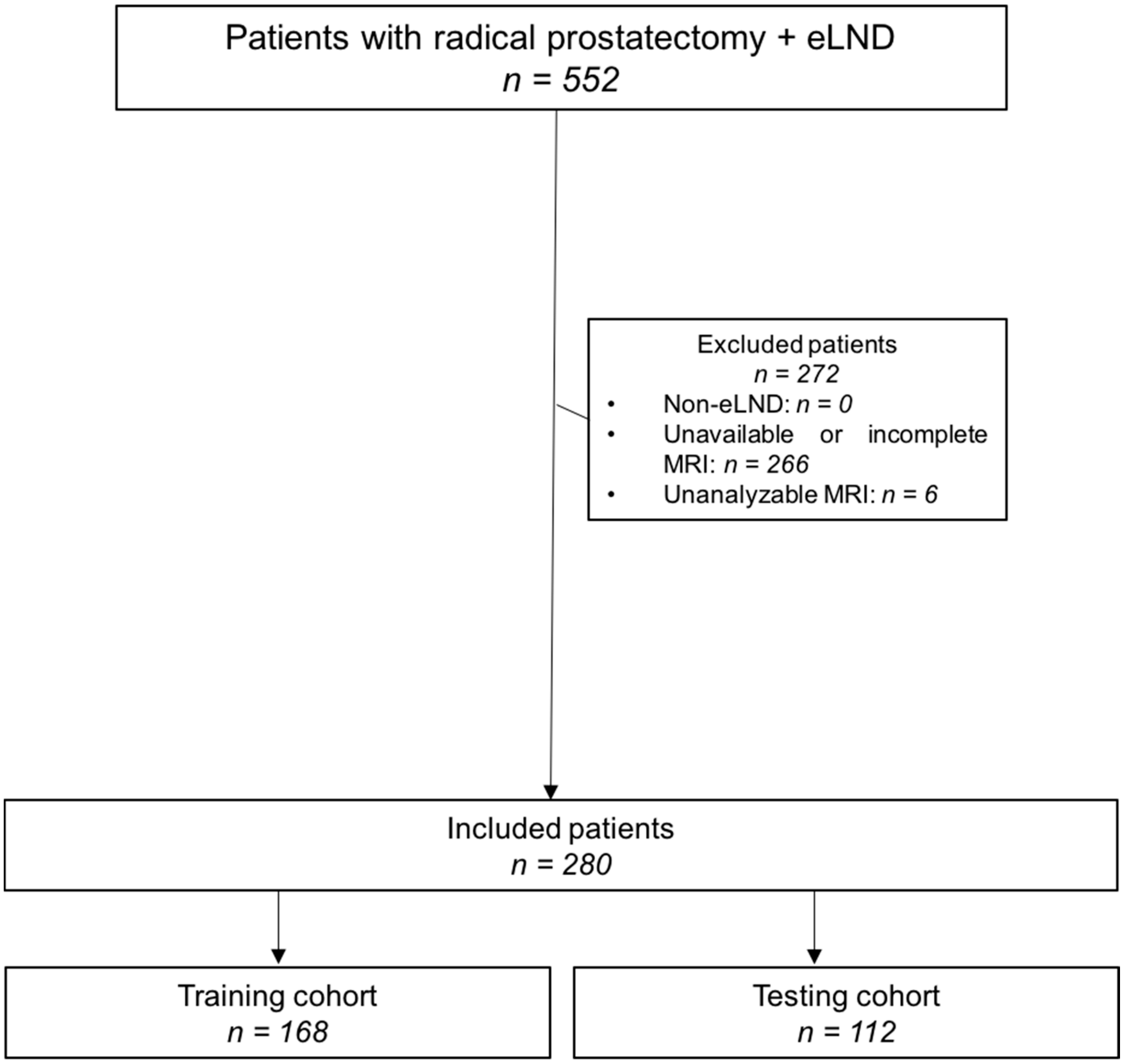

Abbreviations: eLND: extensive lymph node dissection, Non-eLND: non extensive lymph node dissection, MRI: Magnetic Resonnance Imaging

Supplementary Figure 2: Calibration plots for each available model, the New-Combined model and the ComBat-Combined model, in the testing set.

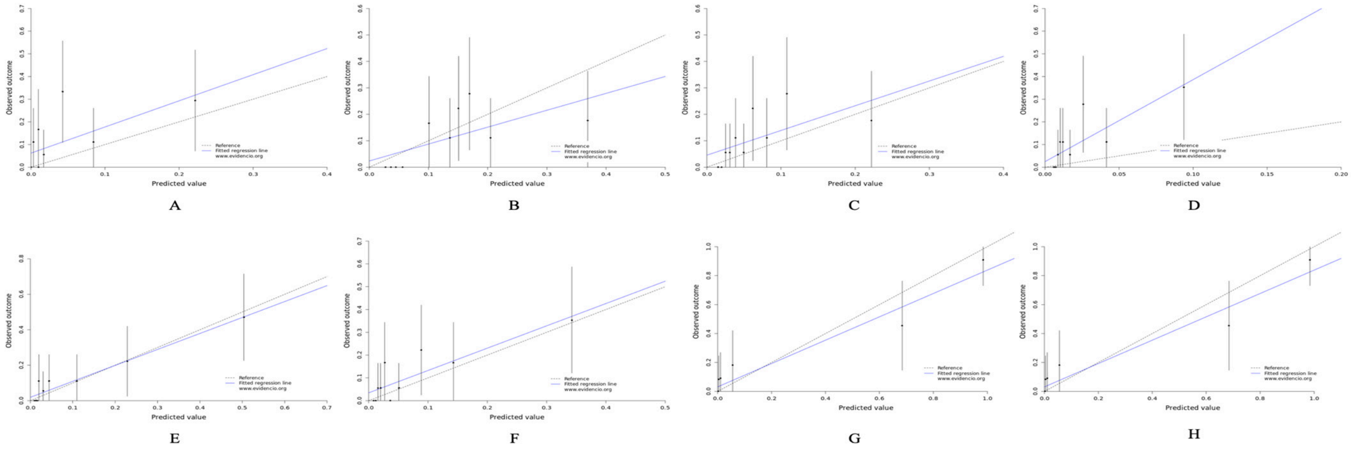

A. Partin
B. Roach
C. Yale
D. Briganti 2012
E. Briganti 2017
F. MSKCC
G. New-Combined
H. Combat-Combined

Supplementary Figure 3: Comparison between the Briganti 2018 and the New-Clinical, the New-Combined and the ComBat-Combined models ROC (*Receiver Operative Characteristics)* curves in the training set (3A) and in the testing set (3B)

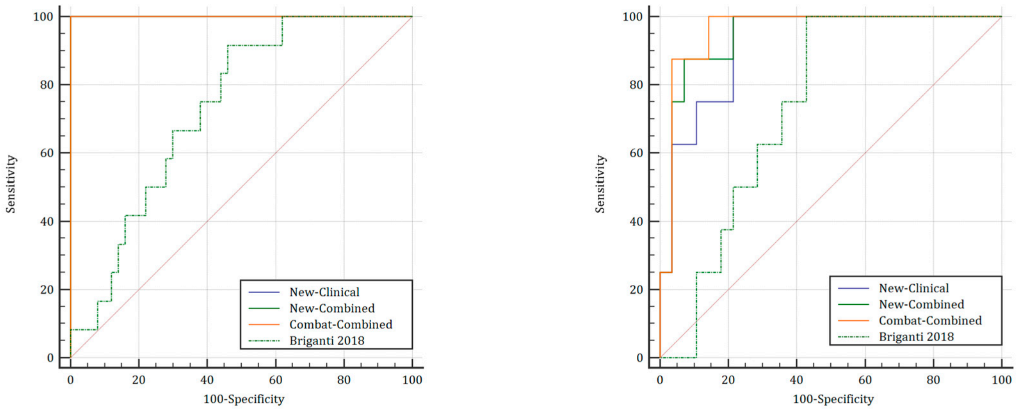

3A

3B

Supplementary Figure 4: Decision curve analysis (A) and calibration plots for the Briganti 2018 (B), the New-Combined model (C) and the ComBat-Combined models (D) in the training set

A

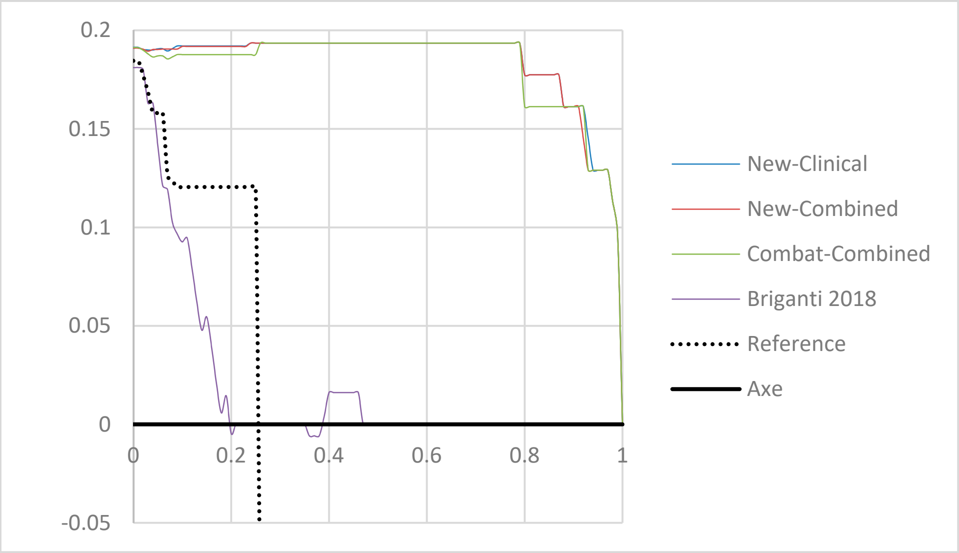

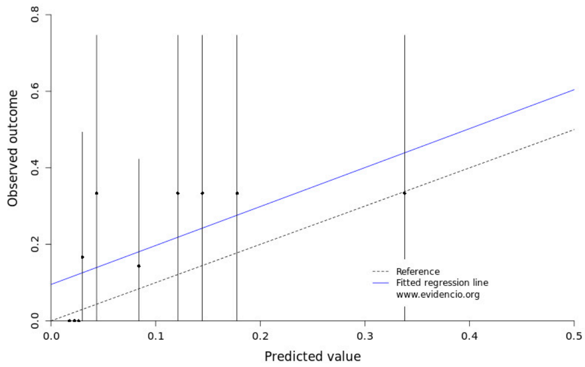

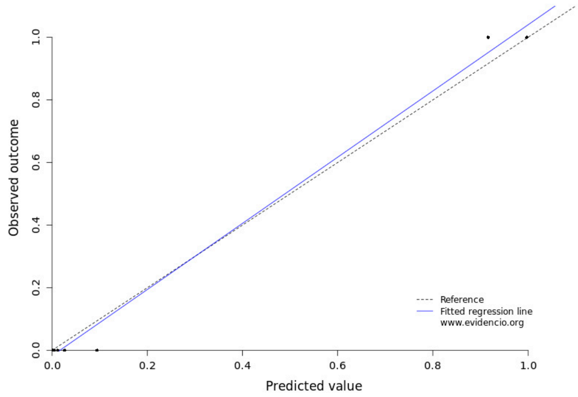

B

C

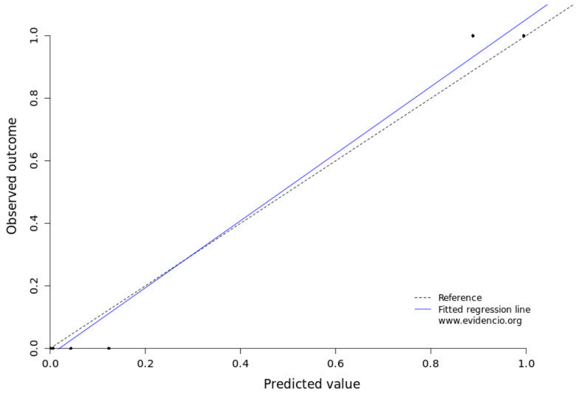

D

Supplementary Figure 5: Decision curve analysis (A) and calibration plots for the Briganti 2018 (B), the New-Combined model (C) and the ComBat-Combined models (D) in the testing set

A

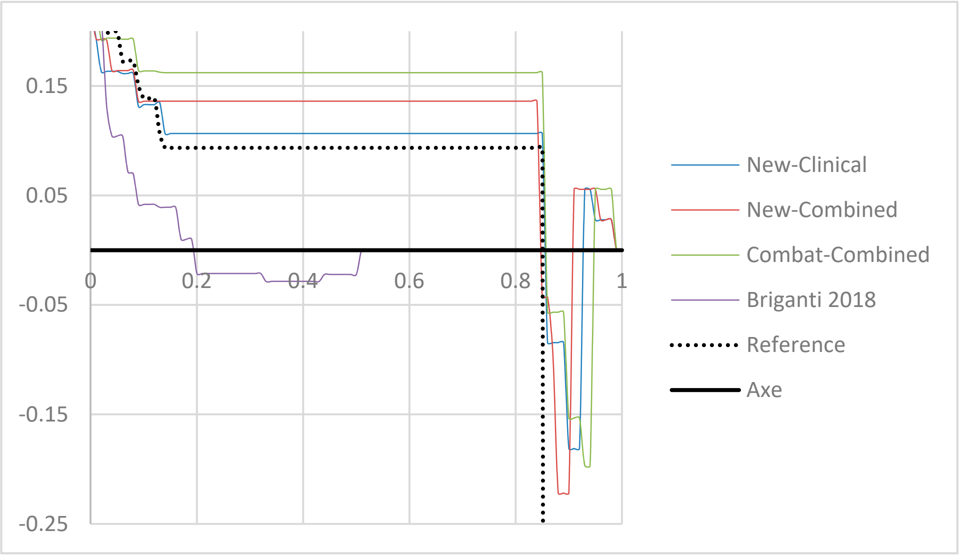

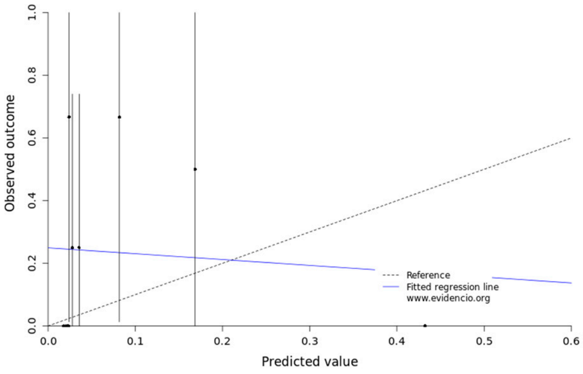

B

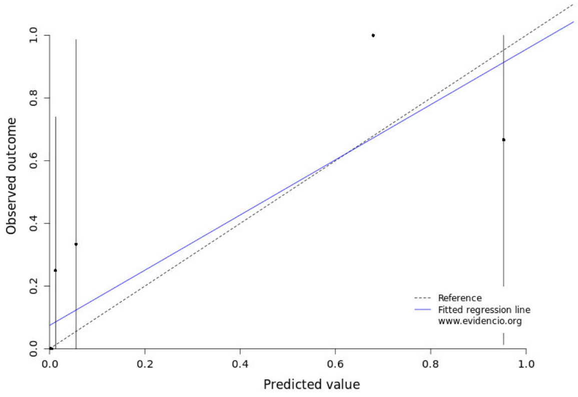

C

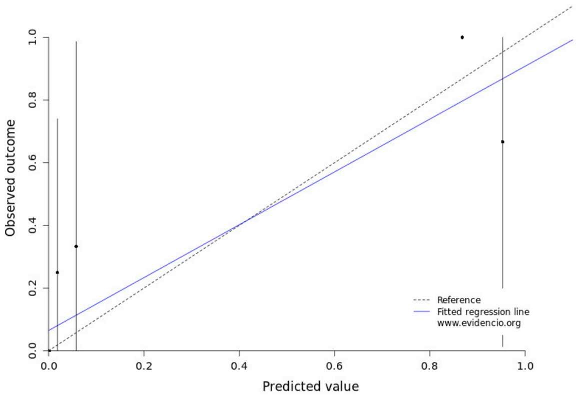

D
